# Objective monitoring tools for improved management of childhood asthma

**DOI:** 10.1186/s12931-024-02817-y

**Published:** 2024-05-03

**Authors:** Phillip L.W. Au-Doung, Jason C.H. Chan, Oliver Y.H. Kui, Marco K.Y. Ho, Yin Ting Cheung, Jenny K.W. Lam, Hak-Kim Chan, John Brannan, Kate C.C. Chan, Albert M. Li, Sharon S.Y. Leung

**Affiliations:** 1 School of Pharmacy, Faculty of Medicine, Chinese University of Hong Kong, Hong Kong SAR, China; 2 Department of Pharmaceutics, UCL School of Pharmacy, University College London, London, WC1N 1AX UK; 3 Sydney Pharmacy School, University of Sydney, Sydney, NSW Australia; 4 Department of Respiratory and Sleep Medicine, John Hunter Hospital, Newcastle, NSW Australia; 5 Department of Paediatrics, Prince of Wales Hospital, Faculty of Medicine, Chinese University of Hong Kong, Hong Kong SAR, China

## Abstract

Asthma is a common chronic disease amongst children. Epidemiological studies showed that the mortality rate of asthma in children is still high worldwide. Asthma control is therefore essential to minimize asthma exacerbations, which can be fatal if the condition is poorly controlled. Frequent monitoring could help to detect asthma progression and ensure treatment effectiveness. Although subjective asthma monitoring tools are available, the results vary as they rely on patients’ self-perception. Emerging evidence suggests several objective tools could have the potential for monitoring purposes. However, there is no consensus to standardise the use of objective monitoring tools. In this review, we start with the prevalence and severity of childhood asthma worldwide. Then, we detail the latest available objective monitoring tools, focusing on their effectiveness in paediatric asthma management. Publications of spirometry, fractional exhaled nitric oxide (FeNO), hyperresponsiveness tests and electronic monitoring devices (EMDs) between 2016 and 2023 were included. The potential advantages and limitations of each tool were also discussed. Overall, this review provides a summary for researchers dedicated to further improving objective paediatric asthma monitoring and provides insights for clinicians to incorporate different objective monitoring tools in clinical practices.

## Introduction

Asthma, one of the most common chronic diseases in children and adolescents [1], is a chronic inflammatory disease with narrowing and inflammation of the small airways in the lungs [2]. It is a non-communicable disease associated with airway hyperresponsiveness to direct or indirect stimuli, such as exercise, exposure to allergens or irritants, change in weather or viral respiratory infection [2]. Symptoms usually begin in preschool age, including recurrent episodes of wheezing, cough, chest tightness, shortness of breath, and difficulty breathing [3]. The symptoms may vary in intensity over time but may also be persistent [3]. Therefore, controlling asthma in children is paramount to ensure their quality of life and wellness [4, 5].

Previous studies showed that poorly controlled and severe persistent asthma could be associated with growth retardation [6] and chronic obstructive pulmonary disease in adulthood [7]. Worryingly, the number of deaths due to childhood asthma worldwide was estimated at 12.9 thousand in 2019 [8]. Therefore, it is essential to ensure that children with active asthma, particularly those with severe asthma symptoms, can be effectively identified and monitored frequently to detect asthma progression and ensure the effectiveness of asthma treatment [9]. However, there are still some unmet needs in asthma control as the rate of admission to the emergency department (ED) and uncontrolled asthma remain high [10–12]. A study from Netherlands estimated that children with a family history of asthma had two-fold higher risk of having uncontrolled asthma [13]. Environmental risk factors including air pollutants nitrogen dioxide (NO_2_), particulate matter with a diameter of 10 microns or less (PM_10_), poor housing condition consists of allergens dust mites or mold are associated with an increased risk of asthma exacerbation and admission to emergency department (ED), particularly in Asia countries [14, 15]. Besides, excessive use of short-acting beta-agonists (SABA) could occur because parents may misunderstand that SABA can control asthma, despite it can only provide quick relief of symptoms but not modify the underlying inflammatory process [16]. Regular monitoring of asthma control may help to identify children at risk of morbidity and mortality. However, the subjective monitoring tools strongly rely on patients’ or their caregivers’ perceptions, while psychological factors may influence their symptom perception, resulting in variation in results and unrealistic reflection of the patient’s actual clinical status [17].

Recently, multiple clinical studies demonstrated the effectiveness of using subjective monitoring tools in conjunction with objective monitoring tools to assess asthma control, such as spirometry (e.g. forced expiration volume in one second, FEV_1_ and forced vital volume, FVC), fractional exhaled nitric oxide (FeNO), bronchial hyperresponsiveness tests [18, 19]. Guidelines have also been updated and recommended that objective monitoring tools, such as spirometry and FeNO measurements, may be used in conjunction to guide asthma preventive treatment in children [20]. However, no gold standard is available, and the tools used in clinical settings differ between guidelines. Therefore, this review aims to summarise the existing objective monitoring tools and their effectiveness in paediatric asthma management.

## Prevalence of asthma in children

The prevalence of childhood asthma in different regions is shown in Table 1 [21–30]. According to the United States (US) National Health Interview Survey 2020, the prevalence was 9.4%, which accounted for about six million children [21]. The United Kingdom (UK) also estimated the prevalence of childhood asthma at around 9%, with one million children receiving asthma treatment [23]. Meanwhile, in the Asia-Pacific region, asthma was more common in developed cities and countries, the prevalence ranged from 2% in China [25] to 10% in Australia [26]. In China, densely populated areas had a higher prevalence rate due to the ongoing urbanisation [25, 31].

When stratifying the prevalence to age and sex, older children and males tend to have a higher prevalence rate. Children aged 10–19 years had a 8.9% higher prevalence rate than children aged 1–9 years in Canada [22]. In Singapore, the prevalence of asthma in children aged 12 to 15 years was 9% higher than in children aged 6–7 years [30]. In addition, the prevalence of asthma in males was 1.9% and 1.6% higher than in females in the US and Australia, respectively [21, 32].

**Table 1:** The prevalence of childhood asthma in different regions

Region	Year	Sample size^a^	Age	Prevalence	Ref
**United States**	2019–2020	NA	0–17	9.4%	[21]
**Canada**	2011–2012	97% of Canadian	1–19	Age 1–9: 10.4% Age 10–19: 19.3%	[22]
**United Kingdom**	2017–2020	NA	0–18	9.1%	[23]
**Europe**	2013	10 million	0–18	9.4%	[24]
**China**	1988–2014	2,678,696	0–14	Overall ~ 2% Densely populated areas had a higher rate, ranging from 0.3% in Tibet to 4.4% in Zhejiang	[25]
**Australia**	2017–2018	~ 4,600,000	0–14	10%	[26]
**Hong Kong**	2001–2002	4,448	6–7	7.9%	[27]
	2001–2002	3,321	13–14	10.2%	[28]
	2021	1,165	6–7	5.5%	[29]
	2021	1,083	13–14	6.1%	[29]
**Singapore**	2001	5,305	6–7	16.3%	[30]
	2001	4,058	12–15	27.4%	[30]

^a^ Overall population of children; NA: Not applicable.

## Severity of asthma in children

While the prevalence of childhood asthma varied among countries, the data suggested that asthma management is suboptimal in many of them, even in developed countries **(**Table 2**)** [21, 23, 28, 33–36]. In the US, nearly 3 million children had one or more attacks in a year [21]. A similar situation was found in Canada, where 65% of asthmatic children had 1–3 asthma episodes in a year [33]. In the UK, nearly half had an attack in the previous year [23]. A European cohort study showed that the severe asthma exacerbation (SAE) rates were the highest in children with severe asthma [34]. In the Asia-Pacific region, 2.1% of children in Hong Kong had an asthmatic attack ≥ 1 time per month [28]. In China, there were 87.7% and 10.1% of children suffered 1–5 and 6–10 attacks per year respectively [35].

**Table 2:** The severity of childhood asthma in different regions

Region	Year	Sample size^a^	Age	Rate of asthma attack	Ref
**United States**	2019–2020	6,856,000	0–17	≥ 1 asthma attack in the past year: 42.7%	[21]
**Canada**	2013–2015	13,367	5–15	Asthma attacks in the past year 1-3 episode: 65% 4–12 episode: 25.4% >12 episode: 9.6%	[33]
**United Kindom**	2017–2020	NA	0–18	Asthma attack in the past year: ~50%	[23]
**Europe**	2008–2013	212,060	5–17	Overall severe asthma exacerbation (SAE) rate 17-198/1000 patient-years (PY) SAE in children with severe asthma: 46–375/1000 PY SAE in children with severe asthma and a history of exacerbation: 283–1000/1000 PY	[34]
**China**	2009 and 2010	10,777	0–14	1–5 attacks per year: 87.7% 6–10 attacks per year: 10.1% > 10 attacks per year: 2.2%	[35]
	2000 and 2010	6,128 and 8 174	0–14	2000: 86% 2010: 77%	[36]
**Hong Kong**	1994 and 1995	4,665	13–14	Severe asthma attack: 6.9% Attack ≥ 1 per month: 2.1%	[28]
	2001 and 2002	3,321	13–14	Wheeze attack past year 1–3 episodes: 6.4% 4–12 episodes: 1.7% > 12 episodes: 0.7%	[28]

^a^ Overall population of children; NA: Not applicable.

Uncontrolled asthma not only contribute to asthma attacks but also cause frequent visits to the emergency department (ED) and hospitalisation **(**Table 3**)** [22, 23, 36–40]. There were 16.6% and 12.5% of children admitted to ED per year in the US and Canada, respectively [21, 22]. In the UK, nearly half of the children aged 0–18 admitted to the hospital for asthma reported having an asthma attack in the past year [23]. The hospitalisation rate decreased by 5% in the United States between 2003 and 2013, in which more younger children (aged 0–4 years old) were admitted to the hospital due to asthma attacks than among those aged 12–17 years [37]. In China, the hospitalisation rate ranged from 16 to 47% [36, 38]. The rate increased slightly in Hong Kong and Singapore between 1996 and 2008 [40]. The asthma control tends to be suboptimal in the Asia-Pacific region because of infrequent monitoring, and the rate of using regular inhaled corticosteroids (ICS) was low [10].

**Table 3:** The hospitalisation rate of childhood asthma in different regions

Region	Year	Sample size^a^	Age	Hospitalisation rate due to asthma attack	Ref
**United States**	2001–2016	NA	0–17	By years 2003: 10% 2013: 5% By age groups Age 0–4: 10.4% Age 12–17: 2.8%	[37]
**Canada**	2015–2016	NA	0–19	Children with asthma admitted to ED: 12.5% (1 in 8) per year Overall asthma hospitalisation rate decreased by 50% from 2006–2007 to 2015–2016: Age 0–4: decreased 50% Age 5–9: decreased 28% Age 10–14: decreased 27% Age 15–19: decreased 23%	[22]
**United Kindom**	2017–2020	NA	0–18	The ED admission rate for children with an asthma attack (per 100,000 children): England: 174 Wales: 165 Scotland: 157	[23]
**China**	2000 and 2010	6,128 and 8,174	0–14	2000: 54% 2010: 47%	[36]
	2019 − 2011	2,960	0–14	ED admission rate: 27% Hospitalization rate: 16%	[38]
**Australia**	2009–2010 2017–2018	NA	0–14	2009–2010: 542 per 100,000 2017–2018: 363 per 100,000	[39]
**Hong Kong**	1996–2008	NA	0–14	Average annual percent change (AAPC) in asthma hospitalisation rate: Age 0–4: 1996–2002: increased 3.6% 2003–2008: increased 4.0% Age 5–14: 1996–2002: decreased 2.2% 2003–2008: increased 2.0%	[40]
**Singapore**	1994–2008	NA	0–14	AAPC in asthma hospitalisation rate: Age 0–4: 1994–2002: decreased 11.8% 2003–2008: increased 1.1% Age 5–14: 1994–2002: decreased 11.3% 2003–2008: increased 1.6%	[40]

^a^ Overall population of children; NA: Not applicable.

### Objective tools in paediatric asthma monitoring

#### Search strategy

The literature search was conducted in two databases, PubMed and Web of Sciences, in July 2023. The search focused on original studies published in English from Jan 2016 to Jun 2023 with available full-text. The following combinations of keywords were used: (“paediatric” OR “children” AND “spirometry” AND “efficacy”); (“paediatric” OR “children” AND “asthma monitoring” AND “FeNO” OR “Fractional Exhaled Nitric Oxide” AND “efficacy”); (“paediatric” OR “children” AND “corticosteroid treatment” AND “FeNO” OR “Fractional Exhaled Nitric Oxide” AND “efficacy”); (“paediatric” OR “children” AND “therapy adherence monitoring” AND “FeNO” OR “Fractional Exhaled Nitric Oxide” AND “efficacy”); (“paediatric” OR “children” AND “guide therapy” AND “FeNO” OR “Fractional Exhaled Nitric Oxide” AND “efficacy”); (“paediatric” OR “children” AND “hyperresponsiveness” OR “bronchial challenge test” OR “bronchial provocation test” OR “Mannitol” OR “exercise” OR “histamine” OR “methacholine” AND “efficacy”); (“paediatric” OR “children” AND “wearable device”); (“paediatric” OR “children” AND “asthma electronic monitoring” AND “efficacy”). The studies were included if they fulfilled the following criteria: children aged ≤ 18 years old and  evaluating the effectiveness in asthma monitoring. Literature reviews, case reports, and studies evaluating the accuracy of objective asthma monitoring tools in comparison with subjective assessments were excluded.

#### Spirometry

Four studies were included, the number of recruited participants ranged from 26 to 612, and the minimum age was 5 years old **(**Table 4**)** [41–44]. The measurement frequency ranged from twice daily to every 3 months [41, 43, 44]. Two studies specified that spirometry was conducted according to the American Thoracic Society and European Respiratory Society (ATS/ERS) guidelines [42, 43].

Performing spirometry at each follow-up can help in paediatric asthma monitoring as it can detect the changes in FEV_1_ and FVC after treatment initiation [44]. FEV_1_ value is an independent risk factor for asthma exacerbations, asthmatic children with FEV_1_ value < 60% could have a double risk of asthma exacerbations [45]. Furthermore, spirometry can identify children with airway obstruction but with fewer symptoms [46]. A cohort study showed that spirometry detected 54% of abnormal results in children who reported good asthma symptom control [42]. This highlights the need to use spirometry to assess asthma control as the severity of asthma may be under-recognised [42]. Suboptimal control of asthma may occur if clinicians evaluate and manage children’s condition only based on symptoms [42].

One study in France recruited participants to conduct spirometry with a portable spirometer at home to monitor their asthma [41]. About 73% of participants reported that the device was acceptable [41]. The mean FEV_1_ did not differ significantly, but it detected a significant decrease (> 40%) in FEV_1_ variability and peak expiratory flow (PEF) variability [41]. Participants could be more familiar with the procedures for performing spirometry upon frequent monitoring. Conducting spirometry at home regularly could help to detect the changes in lung function earlier than children who only have spirometry during follow-up in clinics. It may help to provide a clear lung function profile, serving as a guide for clinicians’ judgment. Frequent monitoring may be feasible as parents were generally satisfied with this approach [41].

**Table 4:** Use of spirometry in paediatric asthma monitoring

Study design	Publication year	Region	Age	Sample size	Spirometry techniques	Outcome(s)	Frequency of measurement	Interventions and/or findings	Ref
Retrospective study	2022	France	6–18 years old children with asthma Median: 9.5 years old	26	NA	Asthma control	10 days during which the patient made twice-daily spirometry recordings	Spirometry results are transmitted to a smartphone in a real-time manner. Abnormal results were sent to physicians by email or SMS, then they contacted the patient within 24 h for follow-up. The mean FEV_1_ for the first 15 days vs. the last 15 days (1.49 L/s ± 0.64 vs. 1.48 L/s ± 0.66) did not differ significantly. FEV_1_ variability decreased from a median of 75.6% at baseline to 35.6% (*p* = 0.006).	[41]
Prospective observational cohort study	2020	UK	5–16 years old children with suspected or diagnosed asthma Mean: 10.3 years old	612	In accordance with the American Thoracic Society and the European Respiratory Society (ATS/ERS) standards	Asthma control	NA	54% of children reported good asthma control had abnormal spirometry and/or raised FeNO. 49% of children reported poor asthma control had normal spirometry and FeNO.	[42]
Prospective study	2020	Korea	7–12 years old children with asthma Mean ± SD: 9.4 ± 1.5 years old	36	In accordance with ATS/ERS standards	Asthma control	Every 3 months	Children with asthma exacerbation (AE) had a significantly lower baseline FEV_1_/FVC than children without AE (all *p* < 0.05). FEV_1_/FVC < 80% was associated with asthma exacerbation in children regardless of inhalant allergen sensitisation (all *p* < 0.05).	[43]
Prospective study	2016	India	6–12 years old children newly diagnosed with asthma Mean ± SD: 8.7 ± 2.0 years old	32	NA	Asthma control	At six weeks, three and six months	FEV_1_/FVC showed significant improvement at three months.	[44]

^a^NA: Not applicable

#### Fractional exhaled nitric oxide (FeNO)

FeNO is a biomarker of eosinophilic airway inflammation to predict children’s asthma conditions and assess the response to ICS therapy [47]. Its level is high in asthmatic patients with T2 inflammation due to the elevated inducible nitric oxide synthases (iNOS) [47]. FeNO is a simple and non-invasive test that measures nitric oxide levels when the patient breathes out [48]. However, it has limited feasibility in younger children because they are unable to exhale at a standard constant flow rate [47]. The success rate was found to be decreased by 60% for 4-year-old children vs. 10-year-old children [49].

The American Thoracic Society (ATS) provides detailed recommendations on interpreting FeNO measurements in children aged 5–12 [50]. A FeNO level lower than 20 parts per billion (ppb) indicates less likely to have benefits if the dose of ICS therapy is increased in children with asthma symptoms [50]. A FeNO level higher than 35 ppb indicates the likelihood of relapse following withdrawal of ICS therapy in asymptomatic patients with stable asthma [50]. It is also associated with an increased risk of asthma exacerbation. Intermediate FeNO values between 20 and 35 ppb are advised to be interpreted carefully with reference to the clinical context [50].

Recent studies evaluated the effectiveness of FeNO in asthma control, response and adherence to the treatment **(**Table 5**)** [51–62]. Paracha et al. indicated a single high FeNO value did not predict adherence to ICS. It should be used in combination with objective tools like spirometry and subjective tools for asthma symptoms to formulate effective management [62]. The role of FeNO in monitoring treatment response to ICS has also been explored, and the results varied. One randomised controlled trial (RCT) found that FeNO did not reduce the risk of asthma attacks compared to subjective monitoring tools [61]. Meanwhile, Fang et al. combined FeNO and spirometry to guide the ICS and showed a reduction in the risk of asthma attacks [58].

Regarding the effectiveness of asthma control, recent studies have shown inconsistent conclusions. In a study of 200 children, high FeNO levels correlated with increased symptom severity and poor asthma control [56]. Another study from Vietnam indicated low level of FeNO (< 20 ppb) was associated with a risk of uncontrolled asthma after followed up 3 months (odds ratio [OR] 1.7, 95% CI 0.8–3.3, *p* < 0.05) [54]. Interestingly, a combination of FeNO with impulse oscillometry (IOS) significantly increased the specificity for predicting uncontrolled asthma compared with FeNO alone (*p* < 0.01) [57]. However, one study showed no significant difference in FeNO to predict asthma attacks between the uncontrolled and controlled asthma groups in 3 to 6-year-old children [51].

**Table 5:** Use of FeNO tests in paediatric asthma monitoring

Study design	Publication year	Region	Age	Sample size	FeNO technique^a^	Outcome(s)	Findings	Ref
Prospective study	2020	China	3–6 years old with or without asthma	111 Children with asthma (*n* = 79) Healthy controls (*n* = 32)	FeNO standard test guidelines of the ATS and ERS standards	Asthma control	FeNO showed no significant difference between the uncontrolled and control asthma groups (*p* = 0.399). Four IOS indices showed significantly higher levels in participants whose symptoms remained uncontrolled than those in the controlled asthma group.	[51]
Prospective study	2017	Korea	8–16 years old with intermittent or mild persistent asthma Mean: 10.9 years old	201	FeNO standard test guidelines of the ATS and ERS standards	Asthma control	FeNO was associated with increased risks for uncontrolled asthma (Hazard ratio [HR] 1.27; 95% CI 1.09–1.49, *p* = 0.003). Combined use of spirometry and FeNO measurements can improve the predictive risk compared to either measurement alone.	[52]
Prospective longitudinal cohort study	2020	Greece, Germany, Belgium, Poland and Finland	4–6 years old with mild-to-moderate asthma Asthma group (mean ± SD): 5.2 ± 0.7 years old Control group (mean ± SD): 5.1 ± 0.8 years old	233 Children with asthma (*n* = 167) Healthy controls (*n* = 66)	FeNO standard test guidelines of the ATS and ERS standards	Asthma control	Wheezing episodes and days with asthma were associated with increased FeNO values (all *p* < 0.05).	[53]
Prospective observational study	2020	Vietnam	Children > 6 years old who newly diagnosed with asthma Mean ± SD: 9 ± 3 years old	93	FeNO standard test guidelines of the ATS and ERS standards	Asthma control	FeNO < 20 ppb had a risk of uncontrolled asthma in the following 3 months (OR 1.7, 95% CI 0.8–3.3, *p* < 0.05). FeNO > 35 ppb at inclusion had a positive predictive value for asthma control at 3 months (OR 3.5, 95% CI 2.2–5.9, *p* < 0.01). Exhaled NO may have a potential role to predict the control of asthma in short-term follow-up in asthmatic children.	[54]
Prospective cohort study	2021	Thailand	4–15 years old with asthma Mean ± SD: 7.9 ± 3.1 years old	178	FeNO standard test guidelines of the ATS and ERS standards	Asthma control	The correlation between the FeNO and control levels demonstrated a high agreement (accuracy index: 91.6%). FeNO < 35 ppb correlated with better asthma control in paediatric allergic asthma.	[55]
Retrospective study	2021	Israeli	Children with asthma and high FeNO levels (range 36–227 ppb) Mean ± SD: 11 ± 3.6 years old	200	FeNO standard test guidelines of the ATS and ERS standards	Asthma control	High FeNO levels correlate with increased daytime (*p* = 0.013 and nighttime asthmatic (*p* = 0.01) symptoms and poor asthma control.	[56]
Prospective observational cohort study	2022	Taiwan	6–12 years old Median: Both were 9 years old	700 Children with asthma (*n* = 560) Healthy controls (*n* = 140)	FeNO standard test guidelines of the ATS and ERS standards	Asthma control	A combination of FeNO (> 20 ppb) with IOS measure significantly increased the specificity for predicting uncontrolled asthma patients compared with FeNO alone (*p* < 0.01).	[57]
Prospective cohort study	2022	China	6–12 years old with newly diagnosed asthma Intervention group (mean ± SD): 8.1 ± 1.7 years old Control group (mean ± SD): 7.9 ± 2.0 years old	133 Intervention group (*n* = 68) Control group (*n* = 65)	NA	Guided corticosteroid treatments	The ICS dose adjustment guided by FeNO and pulmonary function tests could improve asthma control in children and reduce the risk of acute asthma attacks.	[58]
RCT	2019	US	7–18 years old with high-risk asthma Intervention group: 11.2 ± 2.9 Control group: 10.3 ± 2.5	88 Intervention group (*n* = 46) Control group (*n* = 42)	FeNO standard test guidelines of the ATS and ERS standards	Guided corticosteroid treatments	FeNO did not significantly reduce exacerbations and morbidity (*p* > 0.05).	[59]
Prospective study	2020	Vietnam	6–17 years old children with uncontrolled asthma Group 1 (mean ± SD): 10 ± 4 years old (Followed GINA guidelines) Group 2 (mean ± SD): 11 ± 5 years old (Followed GINA guidelines and FENO modification for ICS titration)	222 Group 1 (*n* = 116) Group 2 (*n* = 108)	FeNO standard test guidelines of the ATS and ERS standards	Guided corticosteroid treatments	FeNO combined with GINA guidelines for ICS titration is useful in reducing the daily ICS dose and treatment cost.	[60]
RCT	2022	UK	6–15 years old with asthma and on ICS and has at least one asthma exacerbation during the 12 months before recruitment Intervention group (mean ± SD): 10.0 ± 2.6 years old Control group (mean ± SD): 10.1 ± 2.5 years old	509 Intervention group (*n* = 255) Control group (*n* = 254)	NA	Guided corticosteroid treatments	The addition of FeNO to symptom-guided asthma treatment did not lead to reduced exacerbations among children with asthma.	[61]
Observational cross-sectional study	2023	UK	5–16 years old with asthma and on regular ICS Median: 10 years old	205	NA	Therapy adherence monitoring	A single high FeNO value did not predict adherence to ICS.	[62]

^a^NA: Not applicable

#### Hyperresponsiveness tests

They are the objective tools to measure airway hyperresponsiveness by triggering bronchoconstriction either directly (inhalation of methacholine or histamine to act on the smooth muscle receptors) or indirectly (such as performing exercise or inhalation of adenosine, mannitol to stimulate the release of inflammatory mediators) [63]. Adenosine challenge test, exercise challenge test (ECT) and mannitol challenge test have been investigated in paediatric asthma control monitoring **(**Table 6**)** [64–67]. The recruited children were aged 2, 4 and 6 years old.

The adenosine challenge test stimulates inflammatory mediators’ release to induce smooth bronchial muscle contraction [63]. Solution with adenosine monophosphate (AMP) was administered by nebuliser up to 200 mg/mL. A positive test is classified as one or more of the following: [1] continuous wheeze detected using a stethoscope; [2] oxygen saturation drops at least 5% from baseline; and [3] an increase in the respiratory rate of 50% or more from baseline [64]. Two studies indicated that the test could monitor the children’s condition via the detection of inflammatory changes, guiding the clinician to step up or down the therapy to reduce the risk of asthma exacerbations [64, 65].

ECT induces airway narrowing by running on a treadmill, followed by spirometry to test the lung function [68]. Children with > 12% decrease in FEV_1_ value were considered to have a positive response [68]. ECT increased exercise-induced bronchoconstriction (EIB) detection rate in children with asthma [66]. In addition, mannitol dry powder (MDP) has been suggested as a potential objective monitoring tool to evaluate ICS therapy [67]. Up to a cumulative dose of 635 mg MDP is administered for the mannitol challenge test. A positive response is classified if the dose of MDP induces a 15% decrease in FEV_1_ (PD_15_) versus the baseline value or a 10% fall in FEV_1_ between two consecutive doses [69]. Alternatively, a negative response is defined when PD_15_ is not noted after the maximum cumulative dose [69]. Karantaglis et al. showed that the PD_15_ value increased significantly after the initiation of ICS treatment and decreased with the presence of nocturnal asthma symptoms [67]. While this study demonstrated the potential of MDP challenge test in guiding ICS therapy, the relatively small sample size (23 subjects) may be too few to extend the findings to general population of paediatric asthmatics. Also, there were 3 patients (13.4%) discontinued the test because of severe general discomfort and an urgent tendency to vomit [67]. Although these adverse events were consistent with the previous published studies [69], more research is required to demonstrate the safety profile of using MDP in paediatric asthma monitoring due to the small sample size. The repeated MDP inhalation and spirometry testing procedures could be a possible cause, particularly for younger children who often need more than one inhalation manoeuvres to administer the high dose MDP capsules (40 mg powder per capsule). Other easier lung function evaluation techniques, such as forced oscillation technique (FOT) and interrupter respiratory resistance (Rint), to couple with the MDP challenge test may be considered in future study [70].

Although few studies investigated the effectiveness of hyperresponsiveness tests recently [62], the use of hyperresponsiveness tests in asthma monitoring is not common in clinical settings as the tests are required to be conducted in clinics that limit their feasibility [71]. Parents may feel hesitant to use the tests as they are aware of the occurrence of adverse effects after the use of pharmacological substances to trigger children’s bronchoconstriction [71]. In addition, the tests are not recommended in NICE and GINA guidelines. Particularly, the NICE guideline states that “do not use challenge testing to monitor asthma control” [72]. More research is required to demonstrate their clinical significance in asthma monitoring.

**Table 6:** Use of hyperresponsiveness tests in paediatric asthma monitoring

Objective tools	Study design	Publication year	Region	Age	Sample size	Outcome(s)	Findings	Ref
**Adenosine challenge test**	Retrospective cohort study	2019	Israel	2–8 years old children suspected with asthma Median: 50.5 months	54	Asthma attack	Significant fewer severe asthma exacerbations after completing the challenge test in children with a positive (from 34 to 9 events, *p* = 0.01) or a negative challenge test (from 16 to 0 events, *p* = 0.01).	[64]
	Prospective cohort study	2019	Israel	2–8 years old children suspected with asthma Median: 53 months	159	IgE levels Eosinophils percentage ED /hospitalisations admission rate	Elevated eosinophils percentage, IgE levels, and high number of ED visits/hospitalisations are independently associated with a positive result.	[65]
**Exercise challenge test** **(ECT)**	Cross-sectional study	2019	Netherlands	6–17 years old children with asthma Mean: 11.6 years old	20	EIB	The sensitivity of paediatricians’ predicted diagnosis of EIB was 84%, with a low specificity of 24%. Recommended paediatricians aware of ECT can help to confirm EIB in children with asthma.	[66]
**Mannitol**	Prospective cohort study	2023	Greece	4–16 years old children with asthma Mean: 9.7 years old	23	PD_15_ FEV_1_ Exercise-induced and nocturnal asthma symptoms Safety	Participants completed mannitol at baseline and after three months of treatment with budesonide ± formoterol. The use of ICS resulted in a significant decrease in bronchial hyperresponsiveness to mannitol. PD_15_ value increased significantly in post-treatment (pseudo-median differences 228.5 mg, 95% CI 4.50–458.15, *p* = 0.04) The PD_15_ values were significantly lower in the presence of nocturnal asthma symptoms (median 490 mg, IQR 122–635 vs. median 635 mg, IQR 635–635, *p* = 0.03). **Safety**: Eight patients: nausea and/or tendency to vomit. Two patients could not cooperate. Three patients terminated the use of mannitol due to severe general discomfort and/or an urgent tendency to vomit.	[67]

#### Use of electronic monitoring devices (EMDs) in paediatric asthma

Four studies were included; the outcomes were mainly the effectiveness in asthma control and adherence to inhalation therapy **(**Table 7**)** [73–76]. EMDs have the potential to increase the adherence rate for patients who used pMDI and nebulizer [73, 75, 76]. A study recruited participants between 6 months and 3 years old children to use the EMDs combined with weekly feedback to parents [75]. Results showed a significant improvement in treatment compliance [75]. In addition, van der Kamp et al. combined a smart inhaler, a handheld spirometer and an electrocardiography device [74]. It detected nearly 90% of children with uncontrolled asthma conditions [74]. The use of EMDs may provide guidance to clinicians in assessing patients’ compliance and adherence, as it can collect objective data [77]. The sensor was connected to a nebuliser or inhaler to measure adherence [73, 75, 76]. The devices detected each time actuation and automatically sent reminders to inform healthcare professionals or caregivers via a smartphone app to alert them if any non-adherence occurred [73, 75, 76]. Two studies included clinical outcomes and showed no significant improvement in lowering the rate of asthma exacerbations [73, 76]. Vasbinder et al. also showed no significant in cost reduction of asthma exacerbations between intervention and control groups [76]. More studies are required to evaluate the clinical outcomes and whether the purchasing cost of EMDs can outweigh the direct and indirect cost of hospitalization or ED admission due to nonadherence.

Furthermore, several issues need to be considered before use. Firstly, more evidence is required to demonstrate the effectiveness regarding the validity and accuracy of the devices over a long-term period, as well as their generalisability to other inhalation devices. Secondly, the devices generally only record actuation but not the inhalation technique. Thirdly, the size and the design of the device could affect patients’ willingness to use it [77]. Fourthly, it could increase the workload of clinicians as they require extra time to examine the electronic monitoring data. Clarifying the responsibility in managing, interpreting and discussing data with patients is recommended [77].

**Table 7:** Use of EMDs in paediatric asthma monitoring

Study design	Publication year	Region	Age	Sample size	Study period	Outcome(s)	Interventions	Findings	Ref
RCT	2021	USA	4–17 years old Intervention group (mean ± SD): 9.3 ± 3.2 years old Control group (mean ± SD): 9.2 ± 3.5 years old	252 Intervention group (*n* = 125) Control group (*n* = 127)	12 months	ED admission and hospitalization rate due to asthma attack Adherence to ICS therapy	The inhaler sensor was attached to ICS and SABA transmitted the data (date and time, number of uses) to smartphone in a real time manner. Physicians received alerts if non-adherence occurred (e.g. the participants missed the dose or used SABA > 4 doses per day).	The intervention groups’ mean daily ICS adherence rate increased from 44.9–52.5% at 12 months of follow-up. After 12 months of follow-up, the adjusted rate of asthma-related ED visits and hospitalisations was significantly greater in the intervention group when compared to the control.	[73]
Prospective observational study	2020	Netherlands	4–14 years old	90 Asthma group (*n* = 60) Healthy controls (*n* = 30)	2 weeks	Asthma monitoring	Monitored with wearable devices, including a physical activity tracker, a handheld spirometer, smart inhalers attached to reliver and ICS, and an ambulatory electrocardiography device to monitor heart and respiratory rate.	Detected 88.9% of children with uncontrolled asthma. Able to detect a significant pre-exercise FEV_1_ difference in children with uncontrolled asthma (82.2 vs. 86.1). The rate of using relievers was higher in uncontrolled asthmatic children (mean frequency of use in two weeks 16.5 vs. 3).	[74]
RCT	2020	China	6 months to 3 years old Intervention group (mean ± SD): 2.2 ± 0.8 years old Control group (mean ± SD): 2.3 ± 1.0 years old	96 Intervention group (*n* = 46) Control group (*n* = 50)	6 months	Adherence to ICS	The device was attached to the budesonide nebuliser, recorded the date and time of every actuation, and automatically sent the data to inform the nurse. The nurse provided feedback to the caregivers weekly according to the adherence rate and reminded them to continue using the ICS.	Adherence was significantly higher in the intervention group than in the control group (80% vs. 45.9%. *p* < 0.001).	[75]
RCT	2016	Netherlands	4–11 years old Intervention group (mean ± SD): 7.8 ± 2.2 years old Control group (mean ± SD): 7.7 ± 2.1 years old	209 Intervention group (*n* = 101) Control group (*n* = 108)	12 months	Adherence to ICS Frequency of severe asthma exacerbations	The device connected to the ICS pressurised metered-dose inhaler (pMDI) and recorded the time and date of the administered ICS dose with SMS reminders.	Improved adherence to ICS in children with asthma in the intervention group (69.3% vs. 57.3%). There was no evidence of better asthma control or fewer asthma exacerbations in the intervention group. There is no statistically significant difference in cost reduction of asthma exacerbations between the intervention and control groups.	[76]

## Conclusion

Paediatric asthma outcomes can be severe and even fatal if not well controlled. Therefore, it is crucial to monitor asthma conditions to initiate appropriate management plans to reduce the risk of acute exacerbations. Although no standardised objective tools are available due to limited evidence demonstrating significant benefits in asthma monitoring, spirometry has been widely used to monitor lung function. However, patients who have normal lung function tests can still have the risk of asthma exacerbation. Findings suggest that FeNO may be effective in this condition as well as assessing the treatment responses. Hyperresponsiveness tests with indirect stimuli, such as MDP, have not been included in the NICE and GINA guidelines, but recent research demonstrates it may have potential benefit in asthma monitoring. In addition, some studies suggest that a combination of objective monitoring tools may be more effective in asthma monitoring.

Furthermore, paediatric asthma management has been extended from clinic to home-based settings by incorporating information technology. Although objective testing is scheduled regularly in current clinical practice, it can only be evaluated in clinical settings at infrequent intervals. EMDs send reminders via smartphone applications to users. They are convenient for patients to monitor at home and may help in the early detection of any abnormality in lung function, as well as increase treatment adherence. However, further research is recommended as limited research evaluates their validity and accuracy.

## Data Availability

No datasets were generated or analysed during the current study.
